# Supplementary material for: Cross-linking mass spectrometry uncovers protein interactions and functional assemblies in synaptic vesicle membranes
Source: Nat Commun. 2021 Feb 8;12:858. doi: 10.1038/s41467-021-21102-w (PMC7870876; doi:10.1038/s41467-021-21102-w)
Supplement: Supplementary file 1 — Supplementary Information [file 41467_2021_21102_MOESM1_ESM.pdf]

SUPPLEMENTAL INFORMATION

Cross-linking mass spectrometry uncovers protein interactions and functional assemblies in synaptic vesicle membranes

Sabine Wittig[1], Marcelo Ganzella[2], Marie Barth[1], Susann Kostmann[1], Dietmar Riedel[2], Ángel Pérez-Lara[2, ‡], Reinhard Jahn[2] and Carla Schmidt[1*]

[1] Interdisciplinary Research Centre HALOmem, Charles Tanford Protein Centre, Institute for Biochemistry and Biotechnology, Martin Luther University Halle-Wittenberg, Halle, Germany.

[2] Department for Neurobiology, Max Planck Institute for Biophysical Chemistry, Göttingen, Germany.

* Correspondence: carla.schmidt@biochemtech.uni-halle.de

‡ Current address: Department of Physical Chemistry, Faculty of Pharmacy, University of Granada, Granada, Spain.

Figures

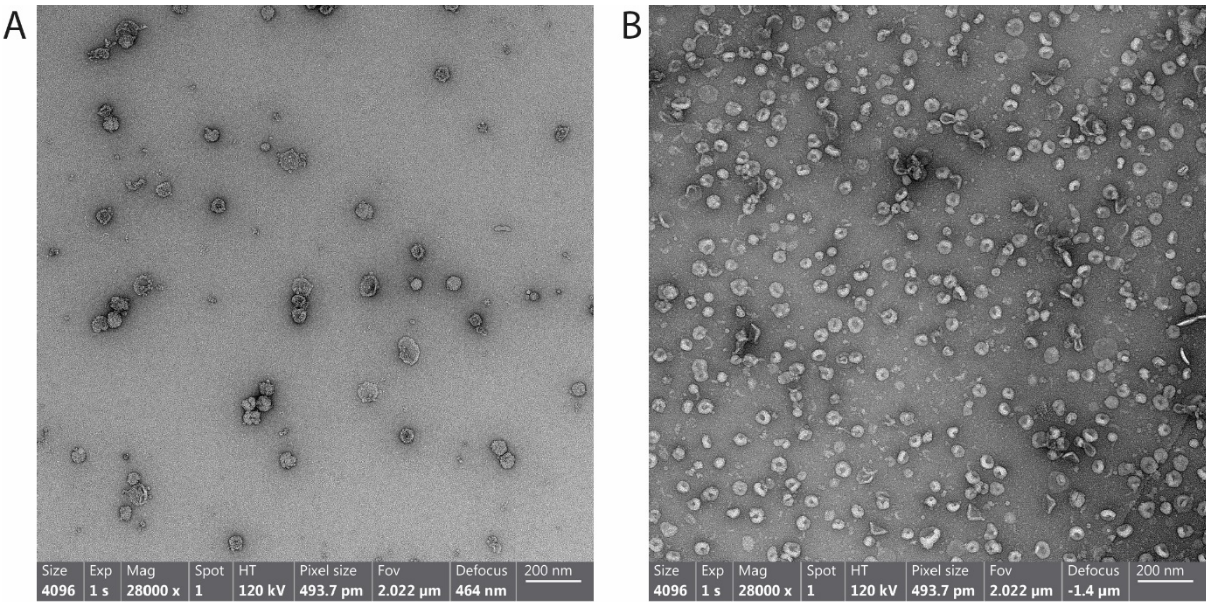

Figure S1: Electron microscopy of purified synaptic vesicles. (A) Purified synaptic vesicles before concentration by ultracentrifugation (n=3). **(B)** Synaptic vesicles after concentration by ultracentrifugation (n=3). See **Methods** for details.

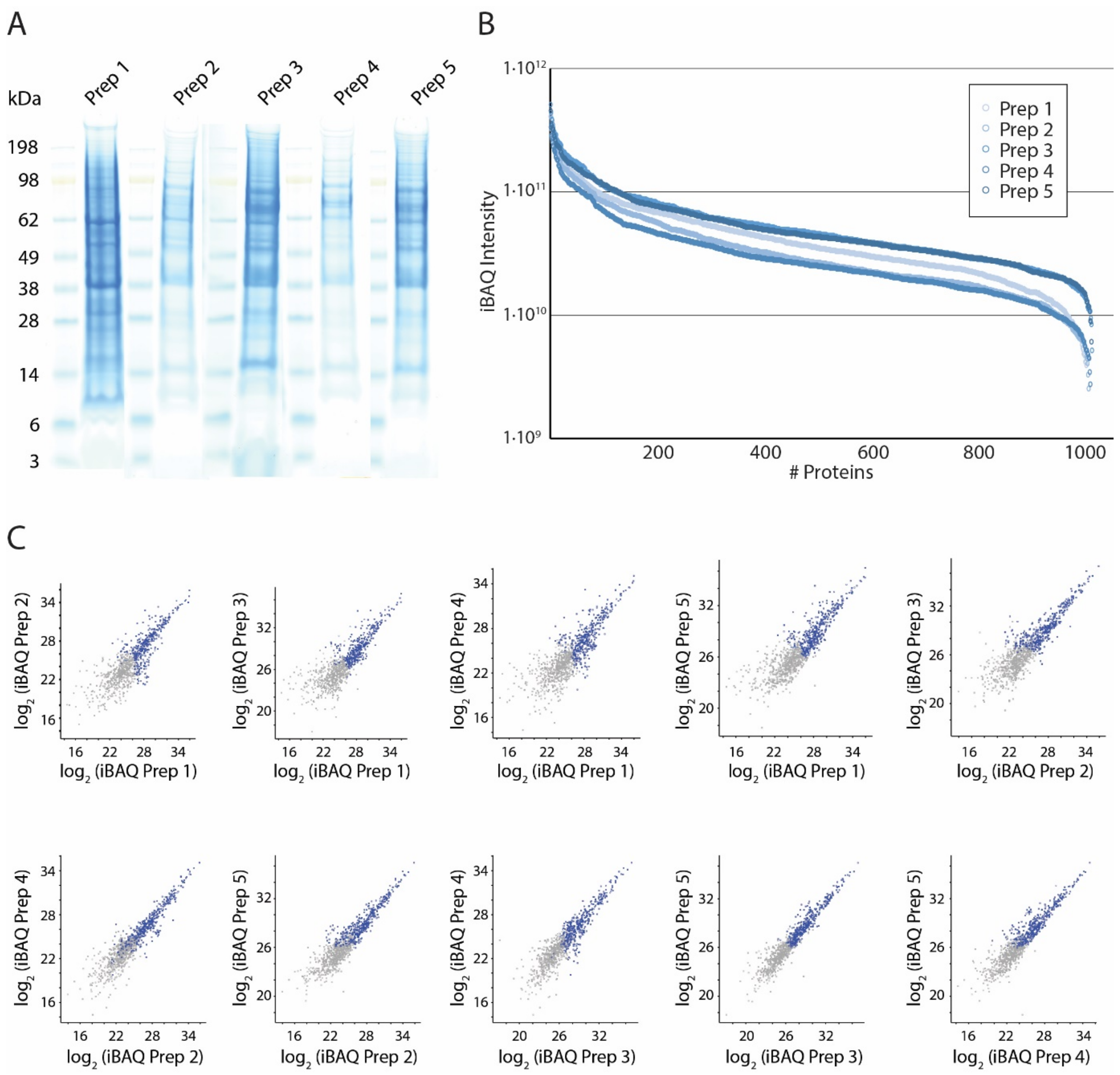

Figure S2: Proteomic analysis of five independent preparations of synaptic vesicles. (A) Gel electrophoresis of the five synaptic vesicle preparations. **(B)** Approx. 1000 proteins were identified with MaxQuant scores >100 in each of the synaptic vesicle preparations. The iBAQ (intensity-based absolute quantification) intensities are plotted for each preparation. **(C)** Pairwise comparison of the five SV preparations. The 400 proteins with highest iBAQ intensities are highlighted in blue.

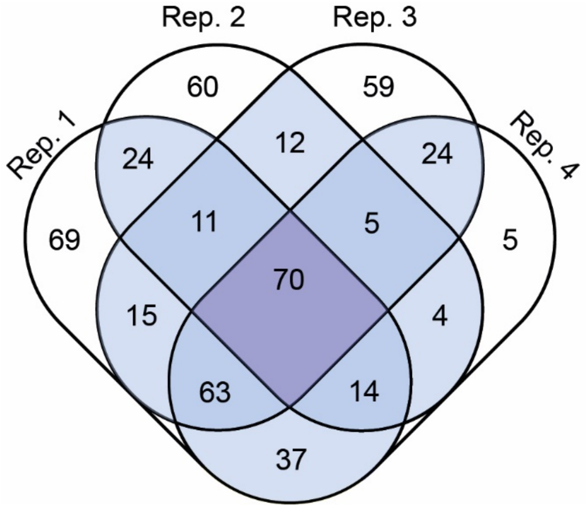

Figure S3: Venn diagram of four cross-linking replicates. A total of 472 cross-links was validated by manual inspection of the mass spectra in four replicates. The Venn diagramme shows the number of cross-links identified in the respective replicates. 279 cross-links (light and dark blue) were identified in at least two replicates. 70 cross-links (dark blue) were identified in all four replicates.

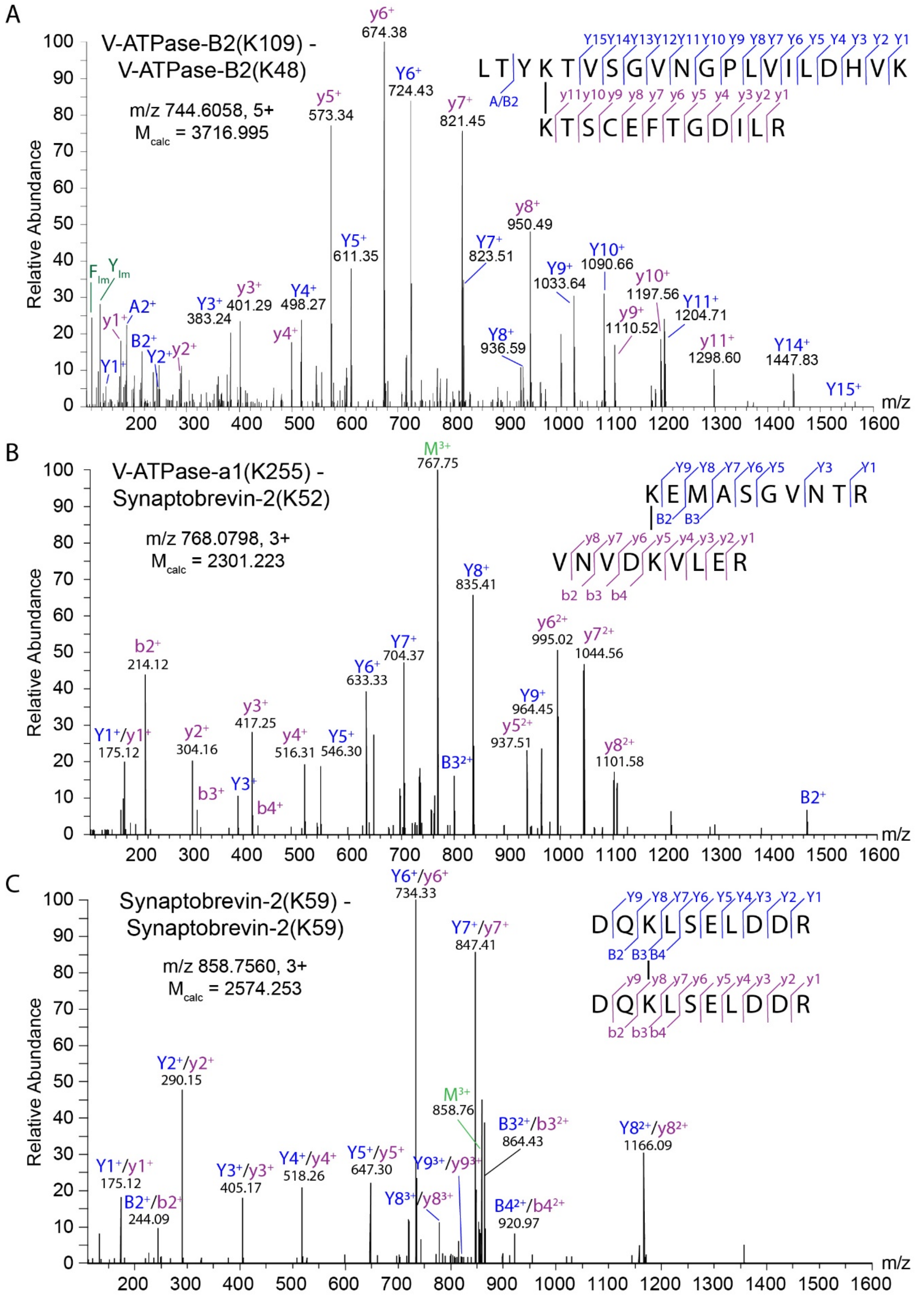

Figure S4: Example spectra of cross-linked di-peptides. B-/b- and Y-/y-ions of the two peptides (blue and purple), the selected precursor (M, green) and immonium ions (IM, green) are assigned. **(A)** Intra-molecular cross-link identified in the V-ATPase subunit B2. **(B)** Inter-molecular cross-link identified between V-ATPase subunit a1 and Synaptobrevin-2. **(C)** Inter-molecular cross-link originating from Synaptobrevin-2 multimers.

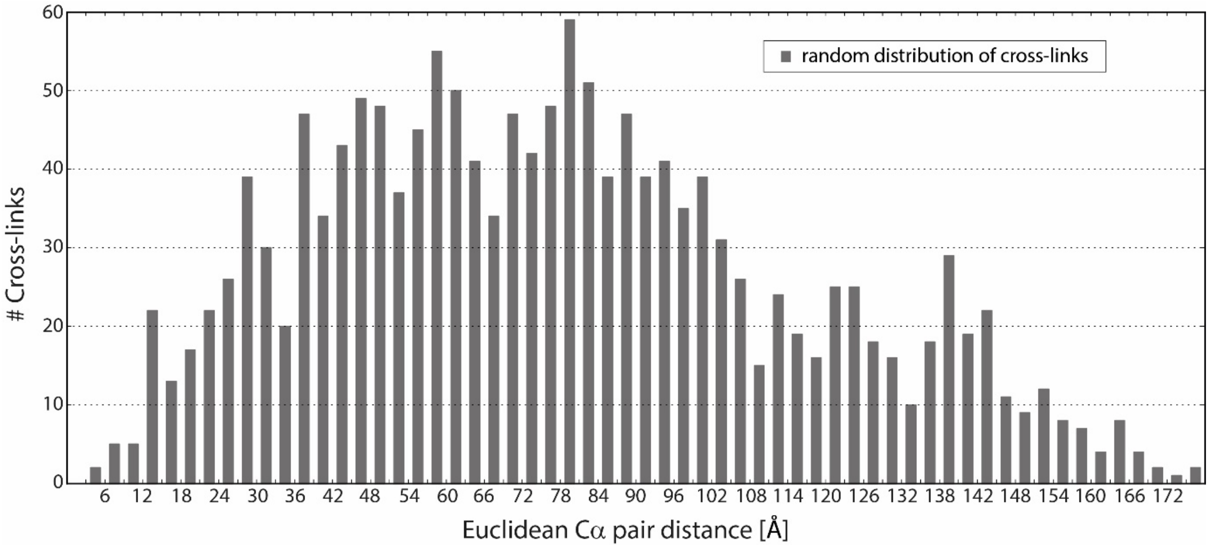

Figure S5: Distribution of random cross-links in the V-ATPase complex. Random cross-linking between identified cross-linked lysine residues was assumed and theoretical cross-linking distances were calculated. The distribution of cross-linking distances of random cross-links is broader and distances are longer when compared with experimentally identified cross-links. See **Figure 2** for distribution of experimentally identified cross-links.

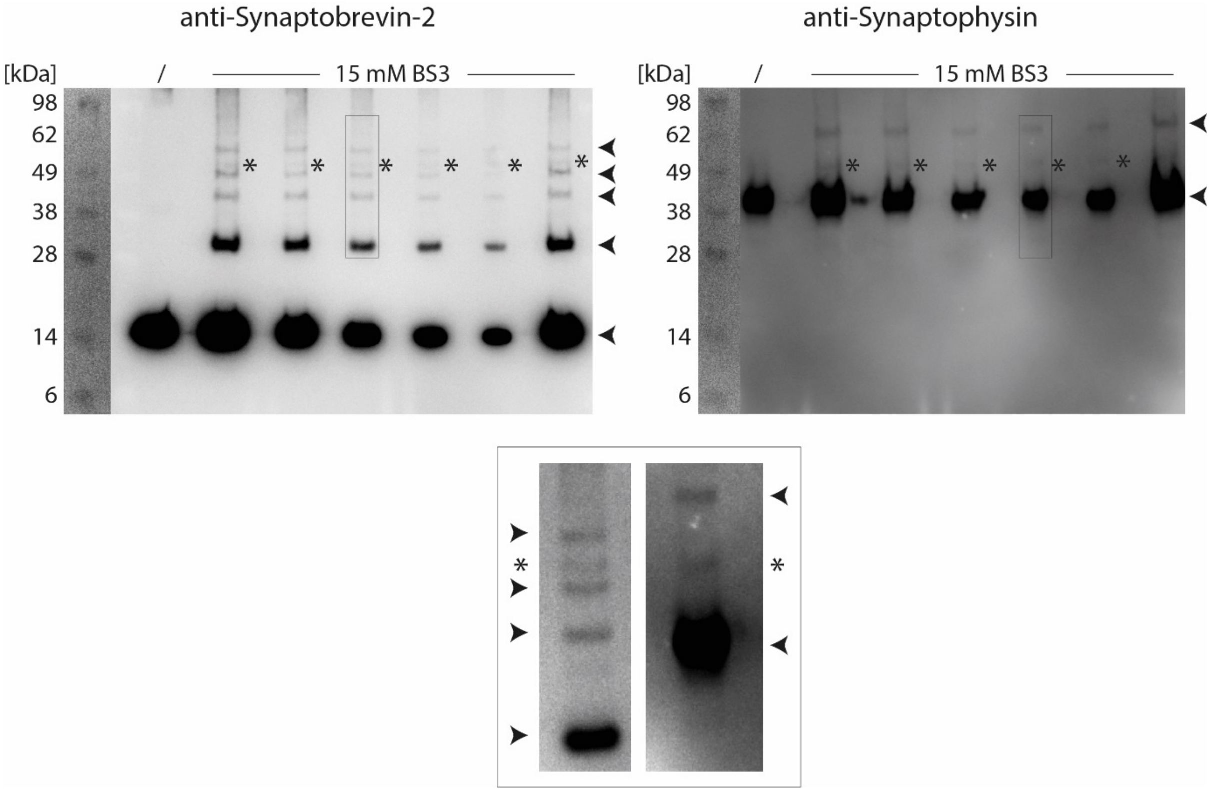

Figure S6: Western blotting of cross-linked synaptic vesicle proteins. Synaptic vesicle proteins were cross-linked with 15 mM BS3. Decreasing amounts of the reaction mixture were loaded onto the gel. Using an anti-Synaptobrevin-2 antibody (lhs), oligomers up to pentamers were observed (indicated by arrowheads). Using an anti-Synaptophysin antibody (rhs), monomers and dimers of Synaptophysin were detected (indicated by arrowheads). An additional band, which was observed with both antibodies (indicated by stars), corresponds in mass to the cross-linking product of the two proteins. The magnification (box) shows this band after background correction of the two blots. n=3

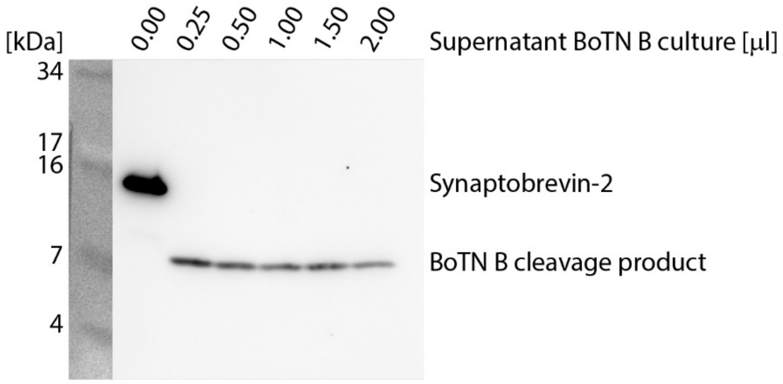

Figure S7: Cleavage of Synaptobrevin-2 with Botulinum Neurotoxin B (BoNT B). Aliquots of synaptic vesicles were incubated with increasing amounts of the supernatant of a BoNT B secreting chlostridium cell culture. Proteins were then transferred to a nitrocellulose membrane and full-length Synaptobrevin-2 (approx. 14 kDa) as well as the BoNT B cleavage product (approx. 6 kDa) were detected using a specific anti-Synaptobrevin-2 antibody. Even at low BoNT B concentrations, Synaptobrevin-2 is completely cleaved. n=3

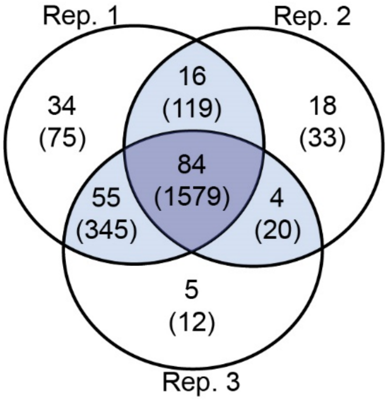

Figure S8: Venn diagram of identified cross-links from three cross-linking experiments after cleavage with Botulinum Neurotoxin B (BoNT B). A total of 216 cross-links was validated by manual inspection of the mass spectra in three replicates. The Venn diagramme shows the number of cross-links identified in the respective replicates. Numbers in brackets correspond to the number of fragment spectra obtained in the three replicates. 159 cross-links (light and dark blue) were identified in at least two biological replicates. 84 cross-links (dark blue) were identified in at least three biological replicates.

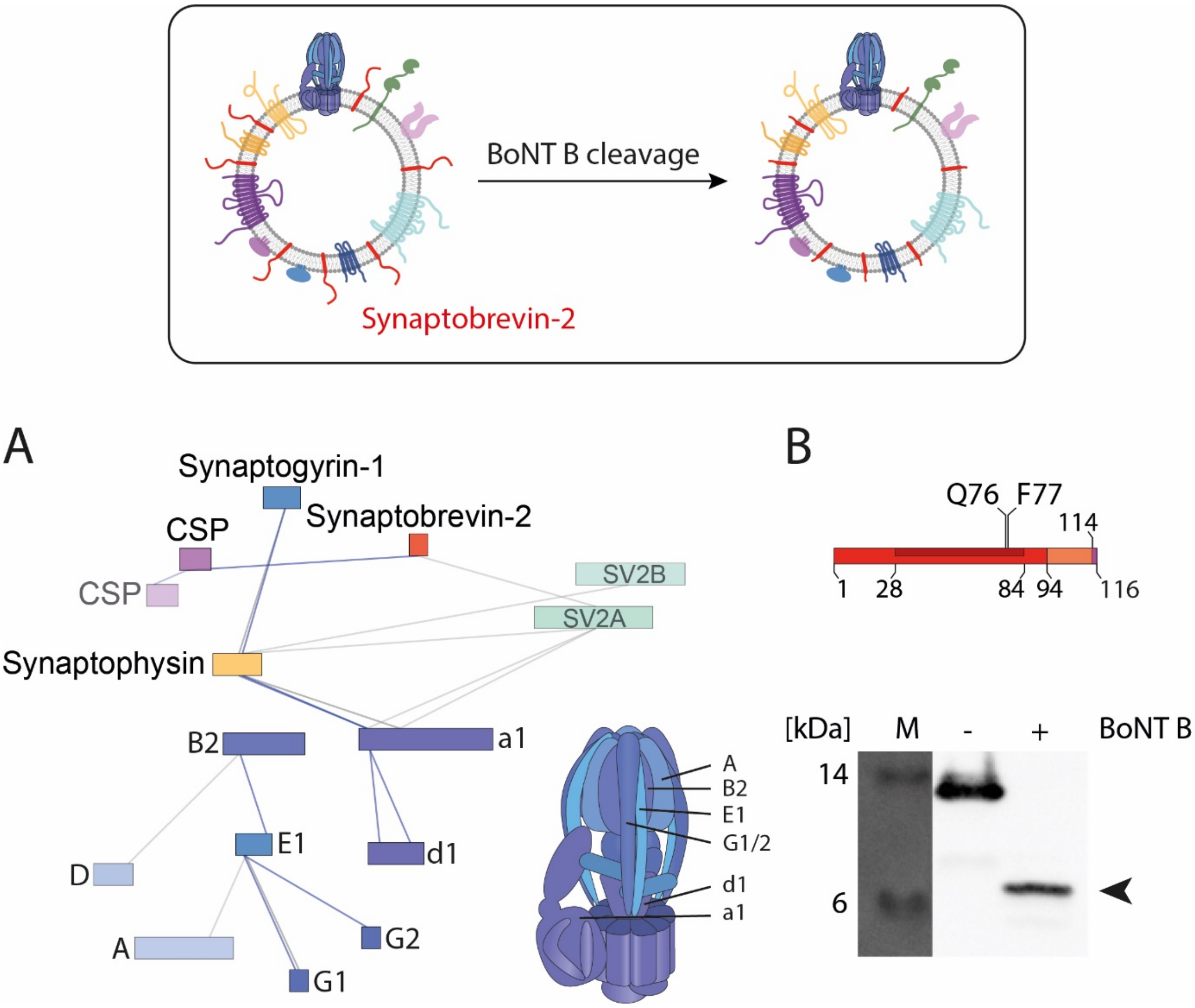

Figure S9: Protein interactions observed after cleavage with Botulinum Neurotoxin B (BoNT B). Top schematic: Synaptic vesicles were incubated with the supernatant of BoNT B producing clostridium bacteria. Synaptobrevin-2 is cleaved by BoNT B. **(A)** Protein network obtained from cross-linking of synaptic vesicles treated with BoNT B. Synaptic vesicle proteins are shown as coloured bars. The length of the bars corresponds to the protein length. The N-terminus is on the left and the C-terminus is on the right side of the bar. Protein interactions that were identified in two or more biological replicates (blue) as well as cross-links identified in only one biological replicate (grey) are shown. Proteins that are linked through interactions from only one biological replicate are transparent. A structural cartoon of the V-ATPase is shown for comparison. **(B)** BoNT B cleavage was verified by western blotting using a specific anti-Synaptobrevin-2 antibody. The intact protein is observed at approx. 14 kDa. After cleavage with BoNT B between residues Q76 and F77 the cleavage product at approx. 6 kDa is observed (arrow head) (n>3). The bar diagramme shows the cytosolic domain (red), the SNARE motif (brown) and the transmembrane domain (orange).

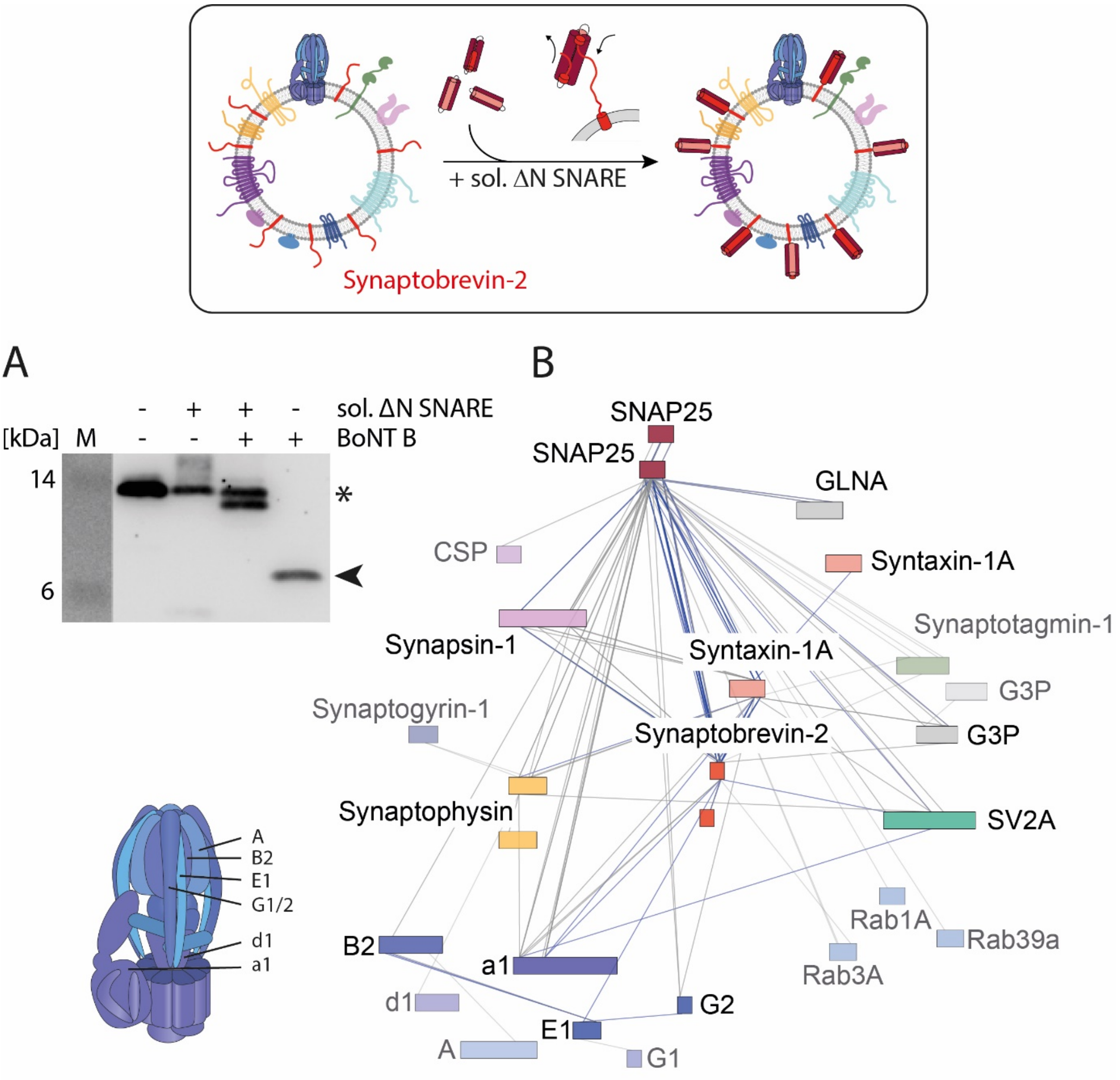

Figure S10: Protein interactions observed after incubation with the soluble ΔN-SNARE complex. Top schematic: Synaptic vesicles were incubated with the soluble ΔN-SNARE complex. Vesicular Synaptobrevin-2 integrates into the ΔN-SNARE complex forming a four-helix bundle. The C-terminal Synaptobrevin-2 peptide (49-96) is released. **(A)** Western blotting confirmed complete incorporation of all Synaptobrevin-2 into the SNARE-four helix bundle. Synaptobrevin-2 assembled into the SNARE complex cannot be cleaved with BoNT B (star). BoNT B cleavage of Synaptobrevin-2 (arrow head) is observed in the absence of the ΔN SNARE complex (n=3). **(B)** Interaction network obtained from cross-linking of synaptic vesicles after incubation with the ΔN SNARE complex. Synaptic vesicle proteins are shown as coloured bars; contaminants are shown as grey bars. The length of the bars corresponds to the protein length. The N-terminus is on the left and the C-terminus is on the right side of the bar. Protein interactions that were identified in two or more biological replicates (blue) as well as cross-links identified in only one biological replicate (grey) are shown. Proteins that are linked through interactions from only one biological replicate are transparent. A structural cartoon of the V-ATPase is shown for comparison.

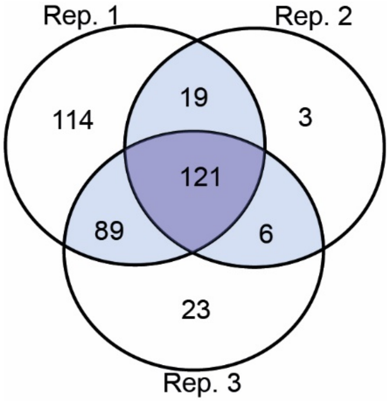

Figure S11: Venn diagram of identified cross-links from three cross-linking experiments after incubation with the soluble ΔN-SNARE complex. A total of 375 cross-links was validated by manual inspection of the mass spectra in three replicates. The Venn diagramme shows the number of cross-links identified in the respective replicates. 235 cross-links (light and dark blue) were identified in at least two replicates. 121 cross-links (dark blue) were identified in three replicates.

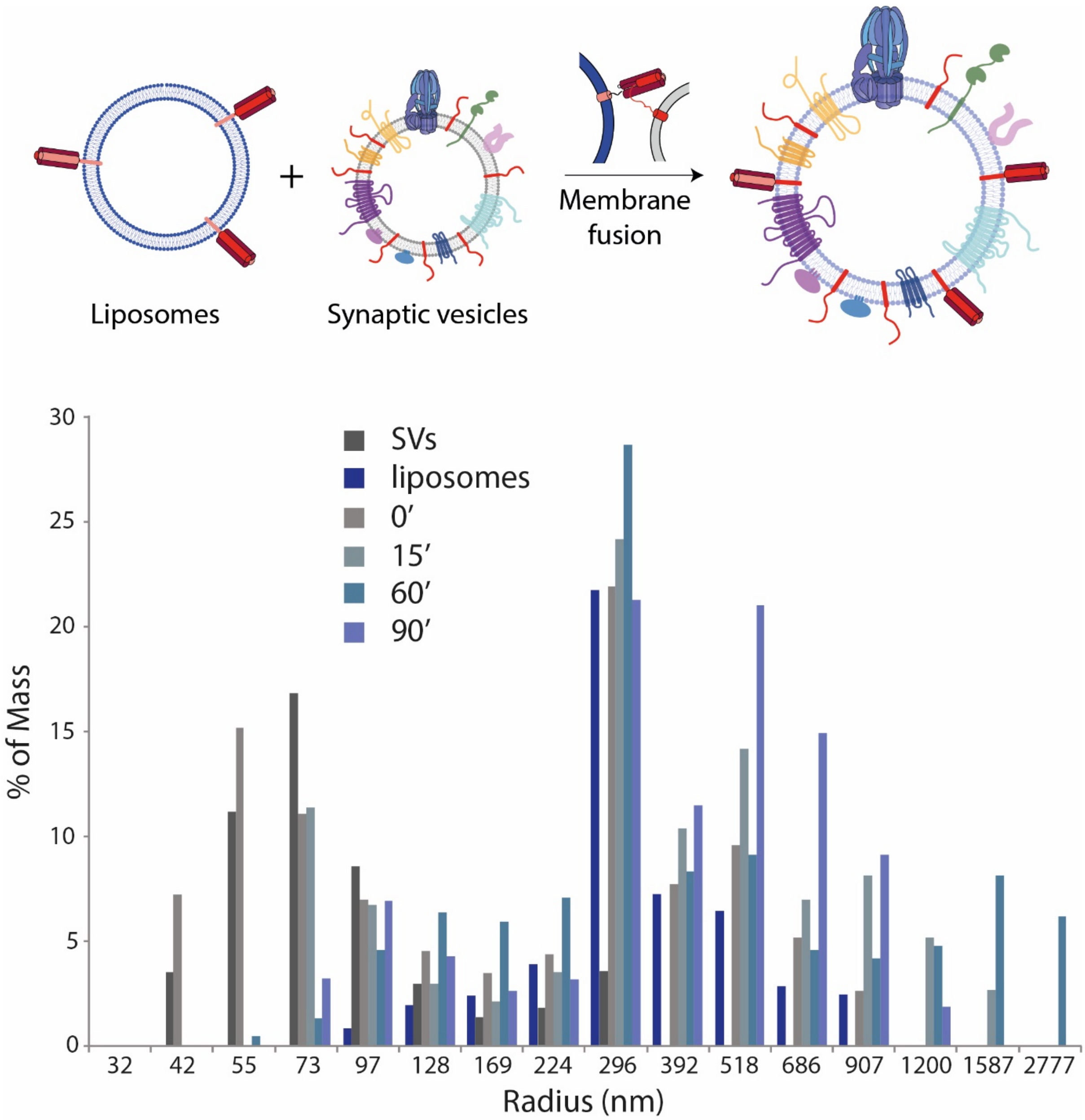

Figure S12: Synaptic vesicle fusion with ‘empty’ liposomes. Top schematic: Synaptic vesicles were fused with ΔN-SNARE proteoliposomes. During membrane fusion, vesicular Synaptobrevin-2 integrates into the SNARE complex. Fused vesicles provide a more spacious membrane environment. Diagramme: Synaptic vesicles (SVs, dark grey) and ΔN-SNARE proteoliposomes (blue) were mixed and incubated. Membrane fusion was followed by dynamic light scattering at different time points (0, 15, 60 and 90 minutes). After 90 minutes a population of fused synaptic vesicles (light blue) of approx. 550 nm was observed. Populations from one biological replicate are shown.

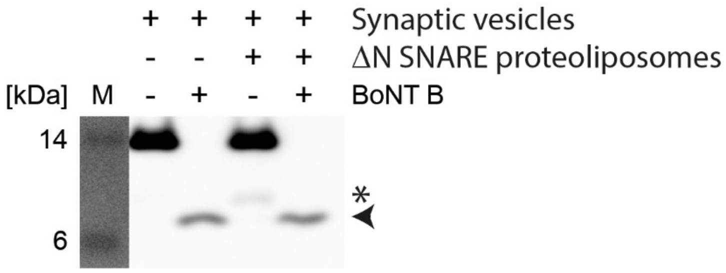

Figure S13: Cleavage of Synaptobrevin-2 with Botulinum Neurotoxin B (BoNT B) after fusion with ΔN-SNARE proteoliposomes. Synaptic vesicles were fused with 'empty' ΔN-SNARE proteoliposomes. Cleavage with BoNT B was employed to prove that only few copies of Synaptobrevin-2 assembled into the SNARE fusion machinery. Full length Synaptobrevin-2 (approx. 14 kDa), the BoNT B cleavage product (arrow head) and the Synaptobrevin-2 fragment originating from the ΔN SNARE complex (star) are detected by western blotting using a specific anti-Synaptobrevin-2 antibody (n=3).

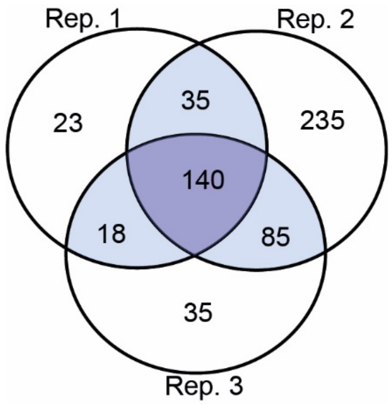

Figure S14: Venn diagram of identified cross-links from three cross-linking experiments after fusion of synaptic vesicles with ‘empty’ liposomes. A total of 571 cross-links was validated by manual inspection of the mass spectra in three replicates. The Venn diagramme shows the number of cross-links identified in the respective replicates. 278 cross-links (light and dark blue) were identified in at least two replicates. 140 cross-links (dark blue) were identified in three replicates.
