# Supplementary material for: Cross-linking mass spectrometry uncovers protein interactions and functional assemblies in synaptic vesicle membranes
Source: Nat Commun. 2021 Feb 8;12:858. doi: 10.1038/s41467-021-21102-w (PMC7870876; doi:10.1038/s41467-021-21102-w)
Supplement: Supplementary file 11 — Reporting Summary [file 41467_2021_21102_MOESM11_ESM.pdf]

nature research

Corresponding author(s): Carla Schmidt

Last updated by author(s): 2020 10 16

Reporting Summary

Nature Research wishes to improve the reproducibility of the work that we publish. This form provides structure for consistency and transparency in reporting. For further information on Nature Research policies, see our Editorial Policies and the Editorial Policy Checklist.

Statistics

For all statistical analyses, confirm that the following items are present in the figure legend, table legend, main text, or Methods section.

n/a	Confirmed	
☐	☒	The exact sample size (*n*) for each experimental group/condition, given as a discrete number and unit of measurement
☐	☒	A statement on whether measurements were taken from distinct samples or whether the same sample was measured repeatedly
☒	☐	The statistical test(s) used AND whether they are one- or two-sided<br>*Only common tests should be described solely by name; describe more complex techniques in the Methods section.*
☒	☐	A description of all covariates tested
☒	☐	A description of any assumptions or corrections, such as tests of normality and adjustment for multiple comparisons
☐	☒	A full description of the statistical parameters including central tendency (e.g. means) or other basic estimates (e.g. regression coefficient) AND variation (e.g. standard deviation) or associated estimates of uncertainty (e.g. confidence intervals)
☒	☐	For null hypothesis testing, the test statistic (e.g. *F*, *t*, *r*) with confidence intervals, effect sizes, degrees of freedom and *P* value noted<br>*Give P values as exact values whenever suitable.*
☒	☐	For Bayesian analysis, information on the choice of priors and Markov chain Monte Carlo settings
☒	☐	For hierarchical and complex designs, identification of the appropriate level for tests and full reporting of outcomes
☒	☐	Estimates of effect sizes (e.g. Cohen's *d*, Pearson's *r*), indicating how they were calculated

Our web collection on statistics for biologists contains articles on many of the points above.

Software and code

Policy information about availability of computer code

Data collection	vendor-specific software for MS instrumentation used (XCalibur v4.2.47, Thermo Scientific), vendor specific software for dynamic light scattering (Dynamics V6, Wyatt Technologies)
Data analysis	Open source software used for data analysis: MaxQuant v1.6.3.3 and v1.6.3.4, Perseus v1.6.2.3 and pLink2.3.9; in-house prepared code for conversion of cross-linking data (CroCo v0.7: Bender, Schmidt 2020); in-house programmed code used for labeling analysis: see provided reference for details (Barth et al 2020); open source software used for cross-link visualization: XVis web server (Grimm et al 2015), XL Analyzer v1.1.4 (Kosinski et al 2015), Chimera v1.14 (Pettersen et al 2004)

For manuscripts utilizing custom algorithms or software that are central to the research but not yet described in published literature, software must be made available to editors and reviewers. We strongly encourage code deposition in a community repository (e.g. GitHub). See the Nature Research guidelines for submitting code & software for further information.

Data

Policy information about availability of data

All manuscripts must include a data availability statement. This statement should provide the following information, where applicable:

- Accession codes, unique identifiers, or web links for publicly available datasets
- A list of figures that have associated raw data
- A description of any restrictions on data availability

All MS raw files were deposited to the ProteomeXchange Consortium (www.proteomexchange.org) via the PRIDE partner repository with the dataset identifier PXD020859. Source data are provided with this paper. For protein identification, uniprot database was employed (taxonomy rattus norvegicus; 31,568 proteins; UniProt proteome ID: UP000002494; 26 Sep 2018). For cross-linking and labelling experiments, a smaller database was generated containing the top 400 protein hits. This database is available together with the raw data at PRIDE with the dataset identifier PXD020859.

Field-specific reporting

Please select the one below that is the best fit for your research. If you are not sure, read the appropriate sections before making your selection.

- [x] Life sciences
- [ ] Behavioural & social sciences
- [ ] Ecological, evolutionary & environmental sciences

For a reference copy of the document with all sections, see nature.com/documents/nr-reporting-summary-flat.pdf

Life sciences study design

All studies must disclose on these points even when the disclosure is negative.

Sample size	No statistical method was used for sample size calculation. Synaptic vesicle preparations include rat brains from 20 animals and purified synaptic vesicles therefore represent an average synaptic vesicle. For all experiments at least 3 biological replicates using different vesicle preparations were performed. An FDR was applied during database searches and, in addition, identified cross-links were manually validated.
Data exclusions	No data excluded.
Replication	All replicates are biological replicates. Protein ID: 5 replicates, Cross-linking SVs: 4 replicates, cross-linking SVs + BoNT B: 3 replicates, cross-linking SVs + DeltaN complex: 3 replicates, cross-linking SVs + liposome fusion: 3 replicates, labelling experiments: 2 replicates for 2 concentrations and two reagents each.
Randomization	Randomization is not relevant to the study because only one sample per experiment was used.
Blinding	Blinding was not relevant to the study because the samples are known and no subjective qualitative results were reported.

Reporting for specific materials, systems and methods

We require information from authors about some types of materials, experimental systems and methods used in many studies. Here, indicate whether each material, system or method listed is relevant to your study. If you are not sure if a list item applies to your research, read the appropriate section before selecting a response.

Materials & experimental systems

n/a	Involved in the study	
[ ]	[x]	Antibodies
[x]	[ ]	Eukaryotic cell lines
[x]	[ ]	Palaeontology and archaeology
[ ]	[x]	Animals and other organisms
[x]	[ ]	Human research participants
[x]	[ ]	Clinical data
[x]	[ ]	Dual use research of concern

Methods

n/a	Involved in the study	
[x]	[ ]	ChIP-seq
[x]	[ ]	Flow cytometry
[x]	[ ]	MRI-based neuroimaging

Antibodies

Antibodies used	anti-Synaptobrevin-2 and anti-Synaptophysin antibodies obtained from Synaptic Systems GmbH: Anti-Synaptobrevin-2 clone 69.1 (Cat.No. 104 211), anti-VAMP1/2/3 (Cat.No. 104 203), anti-Synaptophysin-1/2 (Cat.No. 101 111). Secondary antibodies obtained from Sigma-Aldrich: anti-Mouse IgG (whole molecule)–Peroxidase antibody produced in rabbit (Cat.No. A9044), anti-Rabbit IgG (whole molecule)–Peroxidase antibody produced in goat (Cat.No. A9169)
Validation	All antibodies are characterised. Specification sheets are available at https://www.sysy.com/ and www.sigmaaldrich.com<br>Anti-Synaptobrevin-2 clone 69.1: validated for human, mouse, rat and hamster for applications of WB, IP, ICC, IHC, IHC-P/FFPE, EM and ELISA<br>Anti-VAMP1/2/3: validated for human, mouse, rat and zebrafish for applications of WB, IP, ICC, IHC, IHC-P/FFPE, and ELISA<br>Anti-Synaptophysin-1/2: validated for mouse and rat for applications of WB, IP, ICC, IHC and IHC-P/FFPE<br>Anti-Mouse IgG (whole molecule)–Peroxidase antibody produced in rabbit: validated for mouse for applications of WB, IHC and ELISA<br>Anti-Rabbit IgG (whole molecule)–Peroxidase antibody produced in goat: validated for rabbit for applications of WB, IHC and ELISA

Animals and other organisms

Policy information about studies involving animals; ARRIVE guidelines recommended for reporting animal research

Laboratory animals	Synaptic vesicles were obtained from rat brain (Wistar Kyoto rats, male and female rats at approx. 5-6 weeks age). No animal experiments were performed.

Wild animals	No wild animals were used in the study.
Field-collected samples	No field collected samples were used in the study.
Ethics oversight	Ethical guidelines approved by the Office of Veterinary Affairs and Consumer Protection of the city of Göttingen, Germany. See manuscript for permit number.

Note that full information on the approval of the study protocol must also be provided in the manuscript.
