# Supplementary material for: Cross-linking mass spectrometry uncovers protein interactions and functional assemblies in synaptic vesicle membranes
Source: Nat Commun. 2021 Feb 8;12:858. doi: 10.1038/s41467-021-21102-w (PMC7870876; doi:10.1038/s41467-021-21102-w)
Supplement: Supplementary file 12 — Source Data [file 41467_2021_21102_MOESM12_ESM.pdf]

SOURCE DATA

Cross-linking mass spectrometry uncovers protein interactions and functional assemblies in synaptic vesicle membranes

Sabine Wittig[1], Marcelo Ganzella[2], Marie Barth[1], Susann Kostmann[1], Dietmar Riedel[2], Angel Perez-Lara[2, ‡], Reinhard Jahn[2] and Carla Schmidt[1*]

[1] Interdisciplinary Research Centre HALOmem, Charles Tanford Protein Centre, Institute for Biochemistry and Biotechnology, Martin Luther University Halle-Wittenberg, Halle, Germany.

[2] Department for Neurobiology, Max Planck Institute for Biophysical Chemistry, Göttingen, Germany.

* Correspondence: carla.schmidt@biochemtech.uni-halle.de

‡ Current address: Department of Physical Chemistry, Faculty of Pharmacy, University of Granada, Granada, Spain.

Uncropped Western Blots

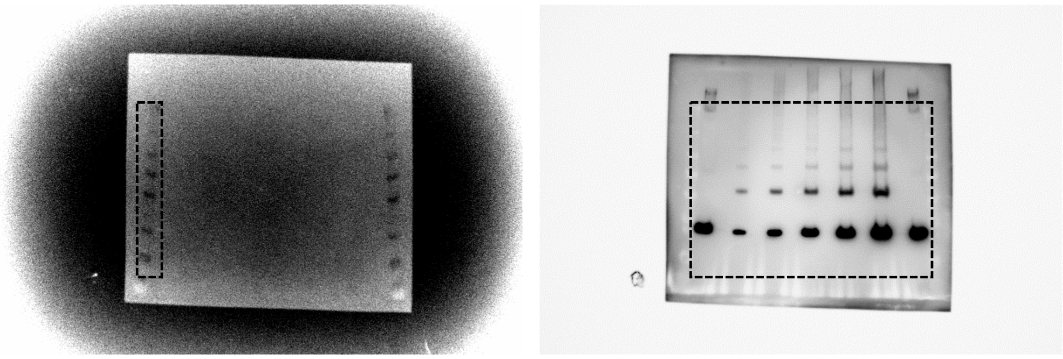

Uncropped Western Blot Figure S6 – anti-Synaptobrevin-2. *Marker (lhs) and anti-Synaptobrevin-2 clone 69.1 (rhs).*

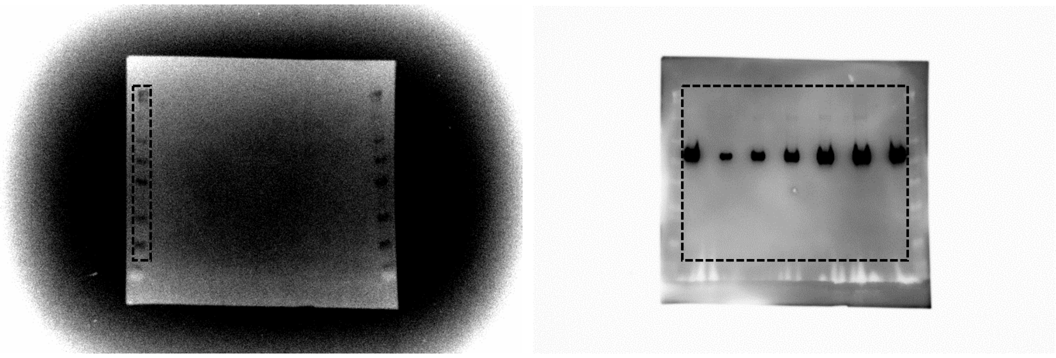

Uncropped Western Blot Figure S6 – anti-Synaptophysin. *Marker (lhs) and anti-Synaptophysin-1/2 (rhs).*

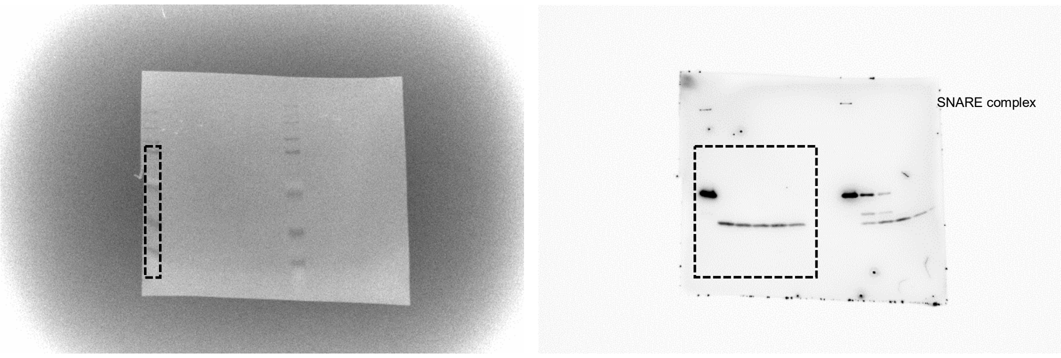

Uncropped Western Blot Figure S7. *Marker (lhs) and anti-VAMP1/2/3 (rhs).*

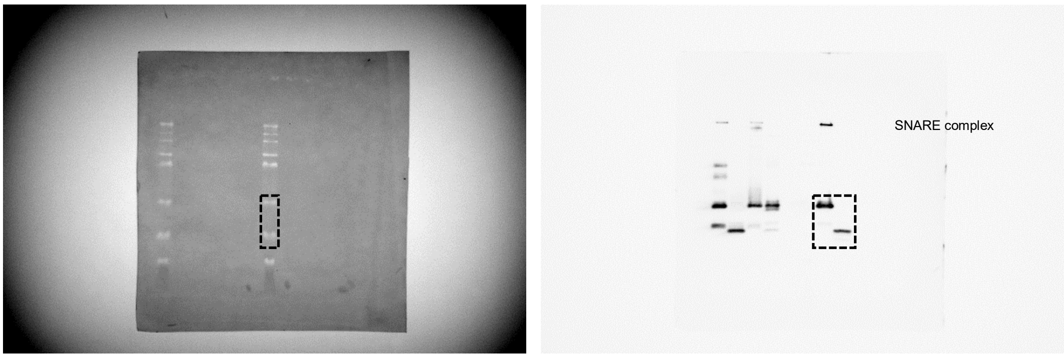

Uncropped Western Blot Figure S9. *Marker (lhs) and anti-VAMP1/2/3 (rhs).*

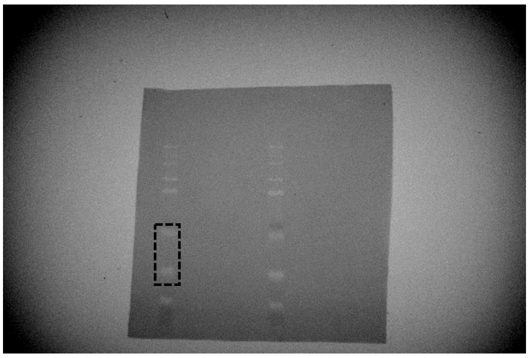

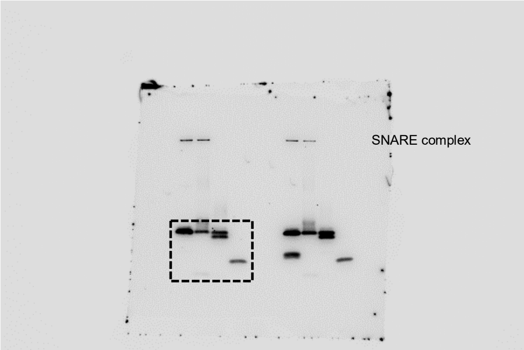

Uncropped Western Blot Figure S10. *Marker (lhs) and anti-VAMP1/2/3 (rhs).*

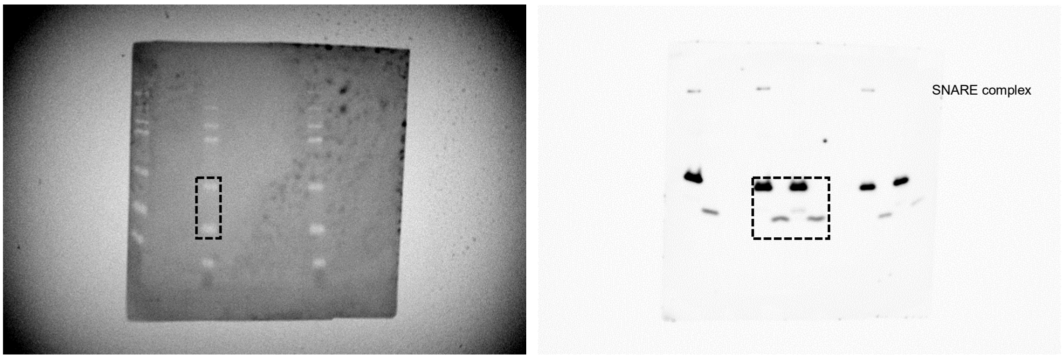

Uncropped Western Blot Figure S13. *Marker (lhs) and anti-VAMP1/2/3 (rhs).*
